# The organization of RNA contacts by PTB for regulation of *FAS* splicing

**DOI:** 10.1093/nar/gku519

**Published:** 2014-06-21

**Authors:** Ian Mickleburgh, Panagiota Kafasla, Dmitry Cherny, Miriam Llorian, Stephen Curry, Richard J. Jackson, Christopher W.J. Smith

**Affiliations:** 1 Department of Biochemistry, University of Cambridge, Downing Site, Tennis Court Road, Cambridge, CB2 1QW, UK; 2 Department of Biochemistry, Henry Wellcome Building, University of Leicester, Lancaster Road, Leicester LE1 9HN, UK; 3 Division of Cell and Molecular Biology, Imperial College, Exhibition Road, London SW7 2AZ, UK

## Abstract

Post-transcriptional steps of gene expression are regulated by RNA binding proteins. Major progress has been made in characterizing RNA-protein interactions, from high resolution structures to transcriptome-wide profiling. Due to the inherent technical challenges, less attention has been paid to the way in which proteins with multiple RNA binding domains engage with target RNAs. We have investigated how the four RNA recognition motif (RRM) domains of Polypyrimidine tract binding (PTB) protein, a major splicing regulator, interact with *FAS* pre-mRNA under conditions in which PTB represses *FAS* exon 6 splicing. A combination of tethered hydroxyl radical probing, targeted inactivation of individual RRMs and single molecule analyses revealed an unequal division of labour between the four RRMs of PTB. RNA binding by RRM4 is the most important for function despite the low intrinsic binding specificity and the complete lack of effect of disrupting individual RRM4 contact points on the RNA. The ordered RRM3-4 di-domain packing provides an extended binding surface for RNA interacting at RRM4, via basic residues in the preceding linker. Our results illustrate how multiple alternative low-specificity binding configurations of RRM4 are consistent with repressor function as long as the overall ribonucleoprotein architecture provided by appropriate di-domain packing is maintained.

## INTRODUCTION

The post-transcriptional steps of gene expression, including splicing and other processing reactions, RNA export, localization, translation and turnover, are controlled by a plethora of RNA binding proteins (RBPs). RBPs interact with their target RNAs via RNA binding domains (RBDs), such as RNA recognition motifs (RRM) and K-homology (KH) domains. The number and RNA-binding specificity of RBDs within individual RBPs varies widely. For example, the RbFox proteins have a single RRM domain that recognizes the specific sequence (U)GCAUG (1). In contrast, many other RBPs contain multiple RBDs, usually with looser specificity. Optimal binding motifs derived by *in vitro* selection methods, such as SELEX (2) or RNA-compete (3), are often degenerate and typically 3–7 nt long, consistent with size of a single RBP-RNA contact. Yet many RBPs have multiple RBDs, so the RNA contacts may consist of more than one short motif. Indeed, attempts have been made to computationally predict targets of multi-RBD proteins by looking for clustered motifs (4,5).

**Figure 1.**
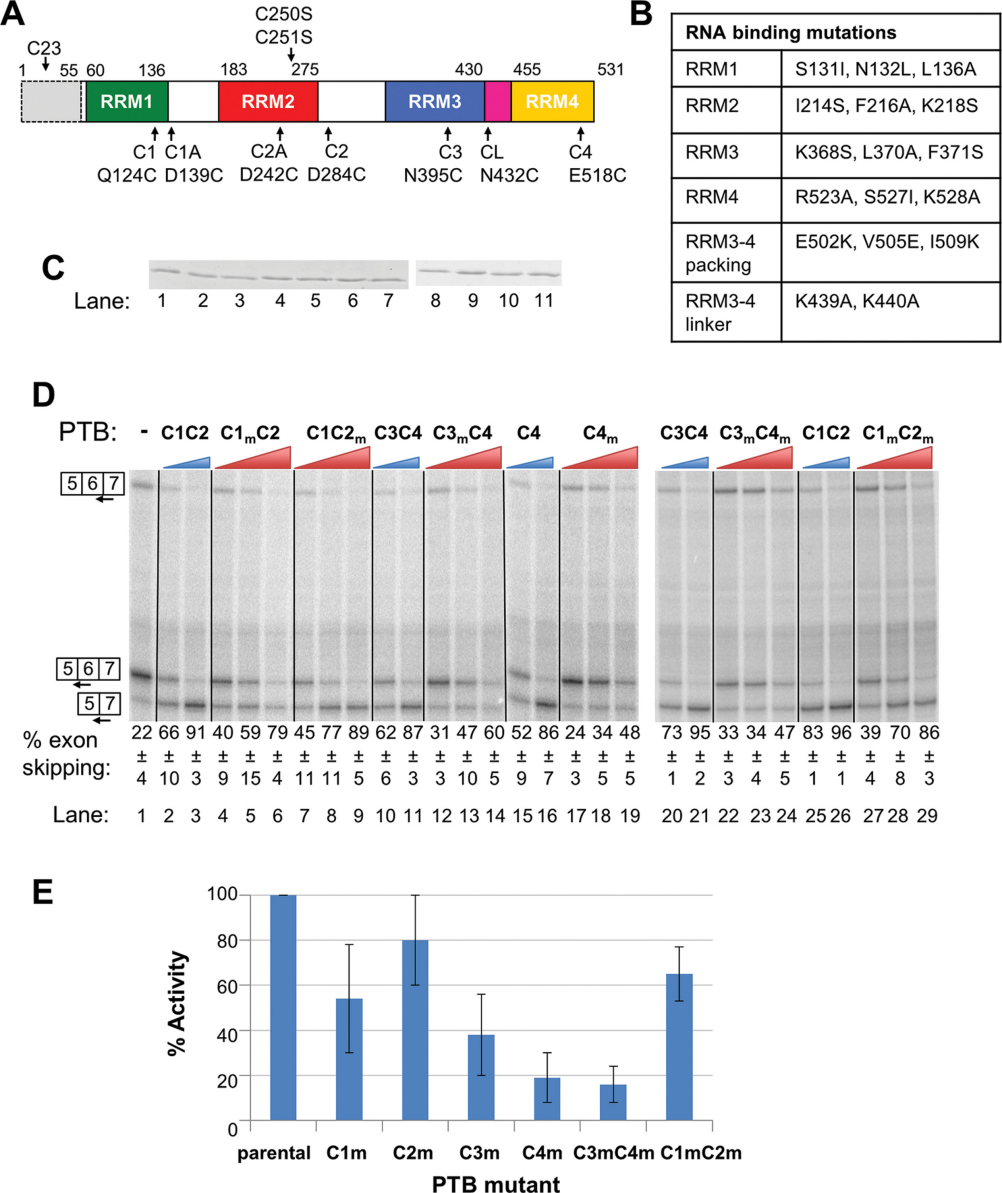
Differential effects of RRM mutations upon PTB activity. (A) Schematic representation of recombinant PTBs used for tethered hydroxyl radical probing. Boundaries of RRMs and positions of cysteines in native PTB1 are shown above. All constructs used here had amino acids 1–55 deleted (shown by grey shading dashed outline) and C250/251 mutated to serine to generate the ‘Cys-less’ parental construct. Positions of mutations to generate single or double cysteine mutants are shown below. All data shown are derived from the C1, C2, C3, CL and C4 mutations, individually and in combination as indicated. (B) Identity of additional mutations designed to impair RNA binding or RRM packing. The mutations in RRMs 1, 2, 3 and 4 were those previously found to be the most effective combinations for inactivating the RNA-binding potential of each RRM (mutation groups m, b, f and k from (18)). Note that the nomenclature used here for the PTB constructs differs from that in (18). (C) Coomassie-stained SDS PAGE of recombinant proteins used in panel D. Proteins are loaded in the same order as in panel D, i.e. lane 1, C1C2… lane 11, C1_m_C2_m_. All proteins migrate at the same size; lanes 1–7 and 8–11 are not adjacent lanes on the same gel. (D) *FAS* WT RNA was spliced *in vitro* in HeLa nuclear extract in the absence (lane 1) or presence (lanes 2–29) of recombinant PTB. PTB was added to 10 and 30 ng/μl (WT single and double cysteine mutants, blue wedges), or 10, 30 and 60 ng/μl (RRM mutants, red wedges). Specific splicing products were detected by primer extension with an end-labeled primer. The percent exon skipping, calculated as 100*5–7/(5–7 +0.5(5–6 + 6–7)), shown below each lane is the average ± SD of three replicates. Proteins are named by the RRM domain(s) containing the cysteine substitutions (C1C2, C3, etc.). The subscript ‘_m_’ indicates an RNA binding mutation (details in panel B) in the associated RRM. For example, C1C2m has the Q124C and D284C substitutions in RRMs 1 and 2, respectively, and the I124S, F216A, K218S RNA binding mutations in RRM2. (E) The % activity of each of the RRM mutants compared to the parental single or double cysteine mutant was calculated for the 30 ng/μl concentrations in panel A: % activity = 100*(%exon skipping mutant PTB − % skipping no PTB) / (%exon skipping WT PTB − % skipping no PTB). For example, for the C1_m_C2 mutant % activity was calculated using the values from lanes 1, 3 and 5 of panel D as 100*(59–22/91–22) = 54%. Error bars are based on the SD of the values in panel D for the mutant and WT proteins.

Polypyrimidine tract binding protein (PTB) has four RRM domains, which mediate binding to pyrimidine rich RNAs, allowing it to regulate splicing, RNA localization, RNA stability and translation (6,7). *In vitro* selection methods show that optimal binding motifs for PTB are mixed pyrimidine sequences, such as UCUU and CUCUCU (3,8). Nevertheless, computational models of PTB binding, trained on CLIP data, indicate that one or more of its RRM domains can also recognize sequences containing G (9), consistent with earlier SELEX data generated under less stringent selection (10). NMR structures of the PTB RRMs bound to RNA show that all 4 RRMs can specifically recognize a CUCUCU ligand (11,12) (Supplementary Figure S1). RRMs 1 and 3 each recognize a core YCU motif, RRM2 recognizes CU, while RRM4 contacts are least specific, with recognition of 5′-YC-3′. The 5′ to 3′ polarity of binding would allow RRMs 1–3 to bind adjacent YCU motifs on unstructured RNA, but the back-to-back di-domain structure of RRMs 3 and 4 necessitates a loop of at least 12 nt between the motifs recognized by each RRM (13). Full length PTB has been refractory to high resolution structural analysis in either free or RNA-bound form, although small angle X-ray scattering indicates an overall extended arrangement of the RRMs (12,14). To circumvent the lack of structural information, tethered hydroxyl radical probing was previously used to interrogate the interaction of the RRM domains of full length PTB with viral internal ribosome entry segment (IRES) RNA (15–17). A series of PTB mutants was created, in each of which a single cysteine residue was strategically placed close to the RNA binding surface of each of the four RRMs (Figure 1A, Supplementary Figure S1). Modification of the cysteines with Fe-BABE allows local generation of hydroxyl radicals which can cleave nearby RNA. Tethered probing analyses indicated the arrangement of PTB RRM binding on EMCV, poliovirus and Aichivirus IRESs (15–17). The four RRMs bound to quite widely dispersed sites on the EMCV IRES, suggesting that PTB-binding might stabilize the 3D IRES structure (15). In contrast, binding to the poliovirus IRES was localized to the base of a single irregular stem-loop, which also binds eIF4G, and PTB binding was found to subtly modulate the orientation of eIF4G binding (16). In subsequent experiments, individual RRMs of PTB were inactivated by targeted mutation, revealing differential RRM-RNA requirements for activity of different IRESs (18).

As highly structured RNAs viral IRESs readily lend themselves to tethered probing, which was developed to map the rRNA sites of contact of ribosomal proteins (19). Tethered probing has also been used to investigate the arrangement of spliceosomal complexes (20–22). However, PTB-regulated alternatively spliced pre-mRNAs represent a particularly challenging subject for such investigation. The PTB binding sites are typically long linear pyrimidine tracts, and in many cases multiple PTB binding events are involved (6). In preliminary experiments we used single-cysteine PTB mutants for tethered hydroxyl radical probing of the *Tpm1* RNA, which we have extensively investigated as a PTB-regulated exon (23–25). However, we saw very few strong contacts, possibly linked with the fact that *Tpm1* binds up to 6 PTB molecules (26), potentially in multiple alternative binding configurations, which might lead to poor signal-to-noise ratios. We therefore chose to investigate a less complex PTB-dependent splicing event.

Regulated skipping of *FAS* exon 6 converts the pro-apoptotic membrane bound receptor to a soluble anti-apoptotic isoform (27,28). A number of splicing regulators have been found to influence this splicing event including PTB, TIA1, HuR, hnRNPC and RBM5 (29–33). In contrast to the majority of PTB-regulated exons, *FAS* pre-mRNA has only one known PTB binding element, the URE6 (U-rich exon 6) within exon 6, which mediates PTB-promoted exon skipping (29). At 16 nt, URE6 is too short to accommodate all four RRMs of PTB (11,34). We therefore anticipated that tethered hydroxyl radical probing should allow us to validate the interaction of PTB with URE6, to clarify which RRMs contact this element, and also to identify additional PTB-RNA contacts at other locations. Here we report the use of a panel of single Cys and RRM mutant PTBs to analyze the interaction of PTB with *FAS* pre-mRNA. Our results indicate that not all RRM-RNA contacts are equal and that the interactions of the intact RRM3-4 di-domain with RNA are particularly important for PTB to regulate *FAS* pre-mRNA splicing.

## MATERIALS AND METHODS

### Reagents

The constructs used for expression of PTB mutants were as described by (15,18). To make *FAS* constructs that could be used to produce the relevant RNAs by T7 RNA polymerase transcription *in vitro*, WT and m0 DNAs were polymerase chain reaction (PCR)-amplified from the respective reporter constructs described in (29,35). The oligonucleotides used for the amplification produced PCR products with EcoRI and BstBI ends, which were used for introduction of the DNAs in the relevant sites of pEMCV-L-VP0 plasmid (15). The use of EcoRI-BstBI restriction sites allowed excision of the EMCV IRES sequence, and its replacement with the FAS DNA sequences. Mutant constructs (URI6m, 5BSsub, 1400sub) (Figure 5) were produced by site-directed mutagenesis from the plasmid bearing the WT FAS sequence. All constructs were verified by sequencing.

**Figure 2.**
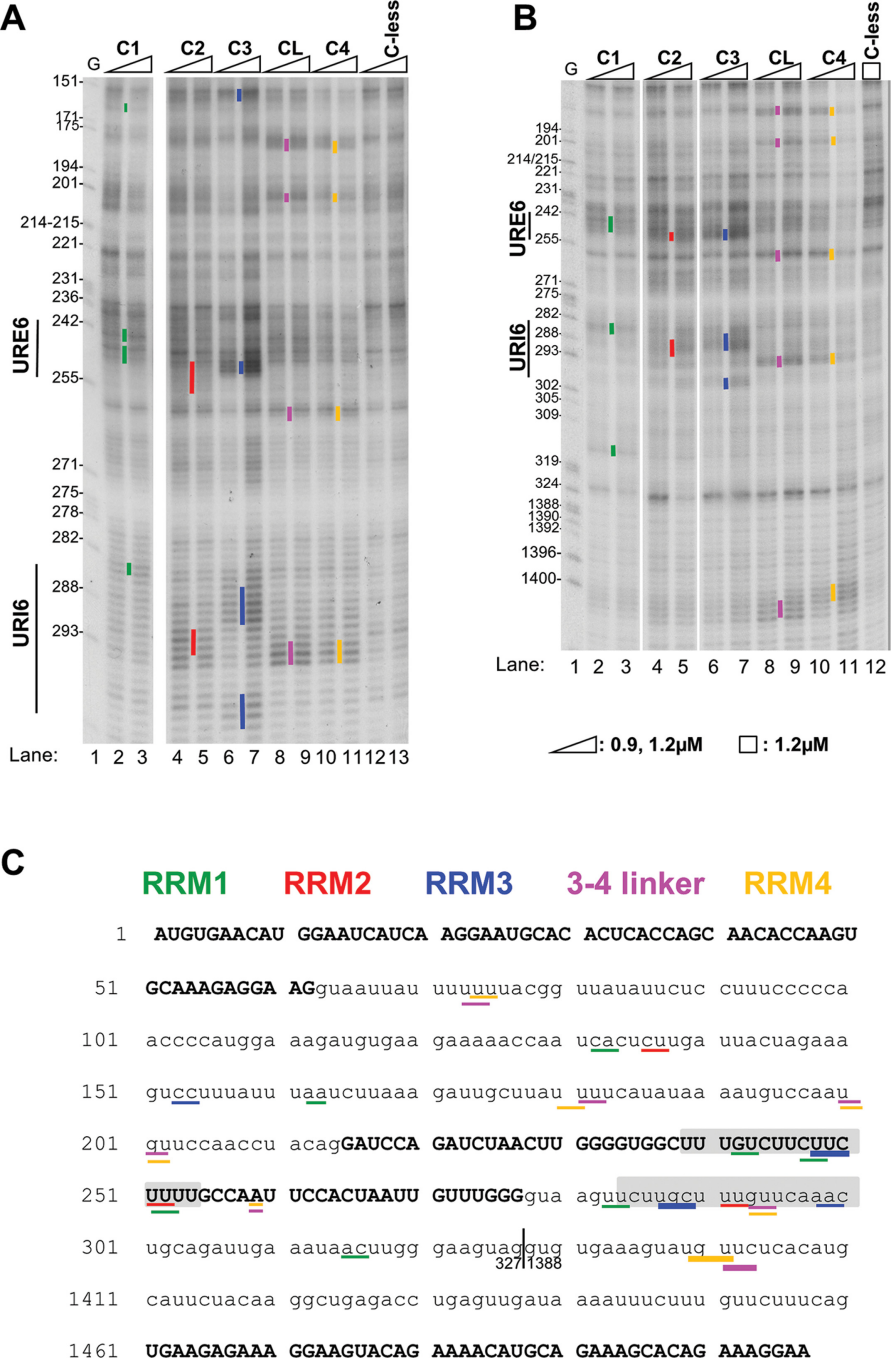
A map of PTB RRM interactions with *FAS* RNA. (A) and (B) Fe(II)-BABE single Cys PTB mutants, as well as the C-less PTB control were used in directed hydroxyl radical probing assays. 0.1 μM of *FAS* WT RNA were incubated with 0.9 μM or 1.2 μM Fe(II)-BABE-PTB mutants. The RNA fragments produced after addition of H_2_O_2_ and ascorbic acid were analysed by primer extension using a set of primers scanning the whole sequence of Fas ΔI6 pre-mRNA. Representative gels, produced by analysis with primer F336R for panel A and F447R for panel B, are shown here. In both panels lane 1 (G) depicts a sequencing ladder generated by the same primer. Cleavage sites are indicated by vertical lines on the left of the corresponding bands on the gel. Mainly the cuts produced by the higher amount of derivatized protein added are indicated. The colour-coding used to indicate the cleavage sites produced by Cysteine residues in the four different RRMs is described in panel C. (C) Summary map of RRM-RNA contacts. The sequence of *FAS* WT RNA is shown. Exons 5, 6 and 7 are depicted with capital letters, whereas lower case lettering is used for intronic sequences. The nucleotides where cleavages were produced in our probing experiments are underlined. Lines of different colours depict cleavage cuts produced by Cysteine residues in the different RRMs as indicated in the inset (green for RRM1, red for RRM2, blue for RRM3, magenta for the linker between RRMs 3–4 and orange for RRM4). Lines of different width indicate the different intensity of the respective band on the probing gels, as this was quantified by SAFA software (described in Materials and Methods). Thicker lines depict strong cleavage bands, whereas the thinner ones correspond to weak cleavage bands. Grey shading depicts the URE6 (nt 239–254) and URI6 (nt 284–300) sequences. The vertical line next to nt 327 shows where the 1060 nt deletion has taken place.

**Figure 3.**
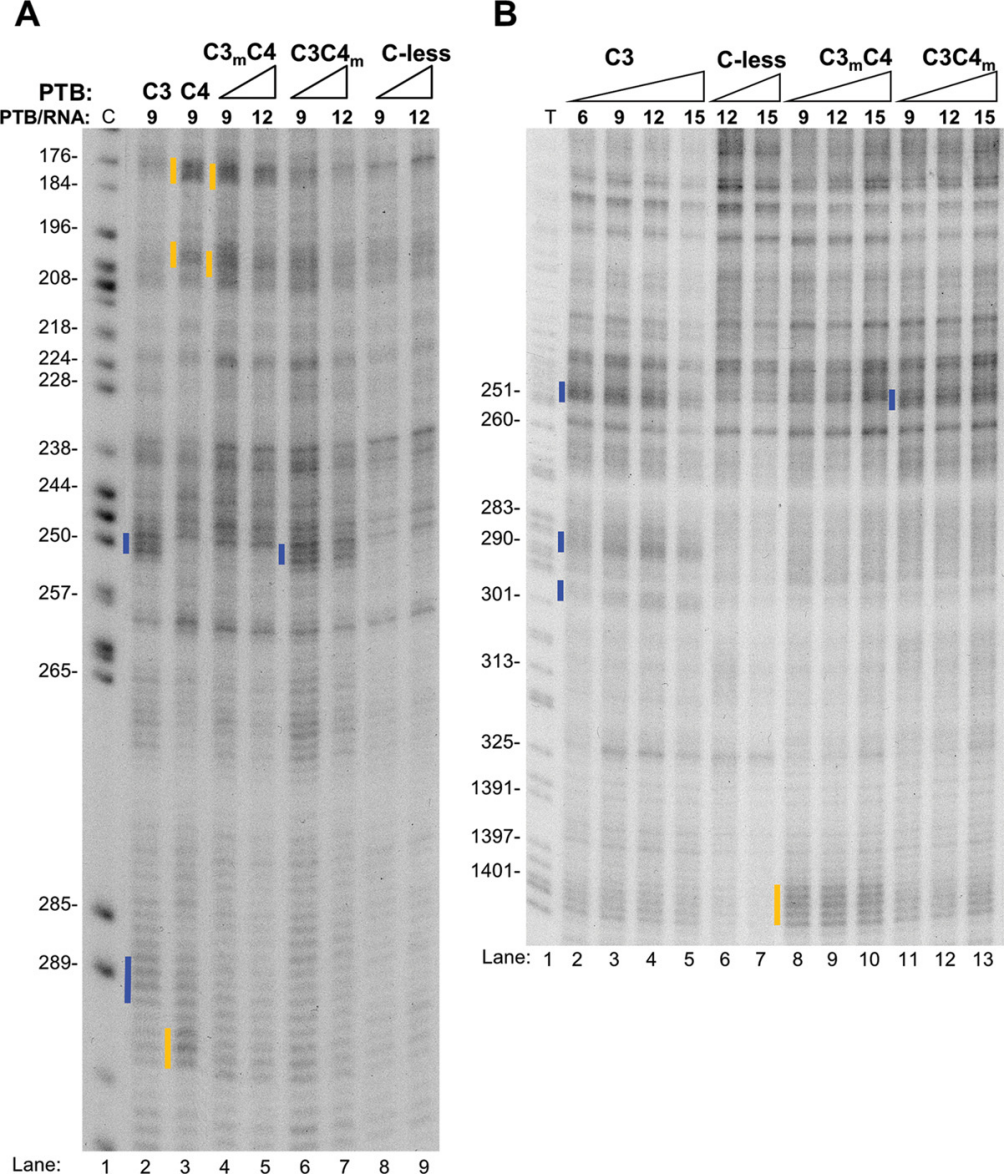
Differential effects of the RNA-binding mutations in RRMs 3 and 4 onto di-domain binding. Fe(II)-BABE Cys PTB mutants were used in directed hydroxyl radical probing assays and analysed as described in Figure 2A and B. The ratio of PTB/RNA used is shown. Cleavage sites are indicated by vertical lines on the left of the corresponding bands on the gel, with the colour-coding introduced in Figure 2. Lanes *C* and *T* depict sequencing ladders generated by the same primer.

**Figure 4.**
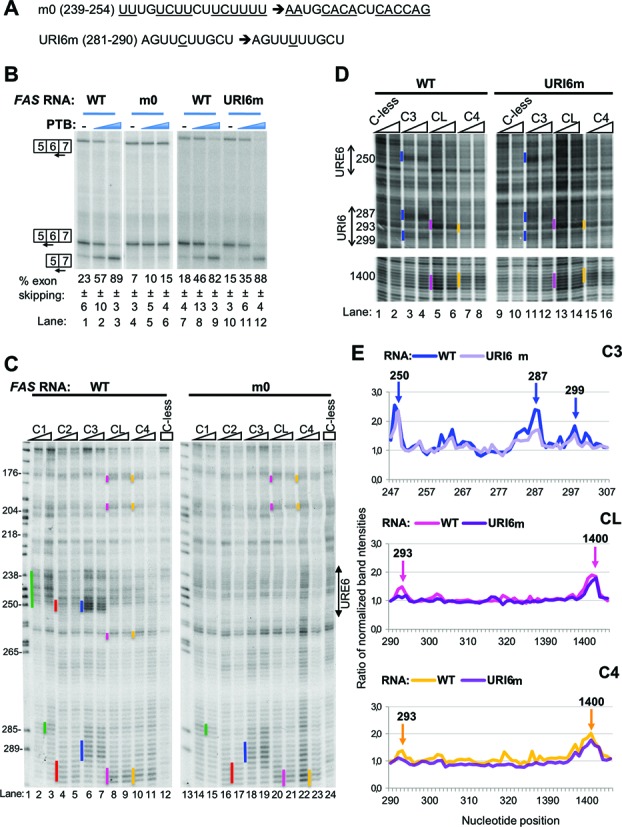
Effects of mutations in URE6 and URI6 elements on PTB's activity in splicing and probing. (A) Sequences of the mutations introduced in URE6 (m0) and URI6 (URI6m) elements of *FAS* RNA. (B) *FAS* WT (lanes 1–3, 7–9) or mutant (m0, lanes 4–6 or URI6m, lanes 10–12) RNA was spliced *in vitro* in HeLa nuclear extract in the absence (−) or presence of recombinant PTB. PTB was added to 10 and 30 ng/μl (blue wedges). Specific splicing products were detected by primer extension with three different end-labeled primers. The percent exon skipping shown below each lane is the average ± SD of three replicates. (C) Fe(II)-BABE single Cys PTB mutants and the Cys-less PTB were used in directed hydroxyl radical probing assays with *FAS* WT (left gel) or m0 mutant RNA (right gel) and analysed as described in Figure 2A. Cleavage sites are indicated by vertical lines on the left of the corresponding bands on the gel, following the colour-coding described in Figure 2. Lane 1 (*C*) depicts a sequencing ladder generated by the same primer. (D) Fe(II)-BABE single Cys PTB mutants and the Cys-less PTB were used in directed hydroxyl radical probing assays with *FAS* WT (left gel) or URI6m mutant RNA (right gel) and analysed as described in Figure 2B. Portions of the gels with the cleavage sites of interest are shown for clarity. (E) Graphs derived from quantification of the gel shown in panel D using the SAFA software, as described in Materials and Methods. After normalization to adjust for small loading variations, the intensity of each band in lanes with an Fe(II)-PTB mutant was compared with the intensity of the same band in the lane loaded with the mock-conjugated Cys-less control. The arrows show the peaks corresponding to the bands indicated by vertical lines in panel D. Numbers above the arrows indicate the exact nucleotide position of the cleavage site.

**Figure 5.**
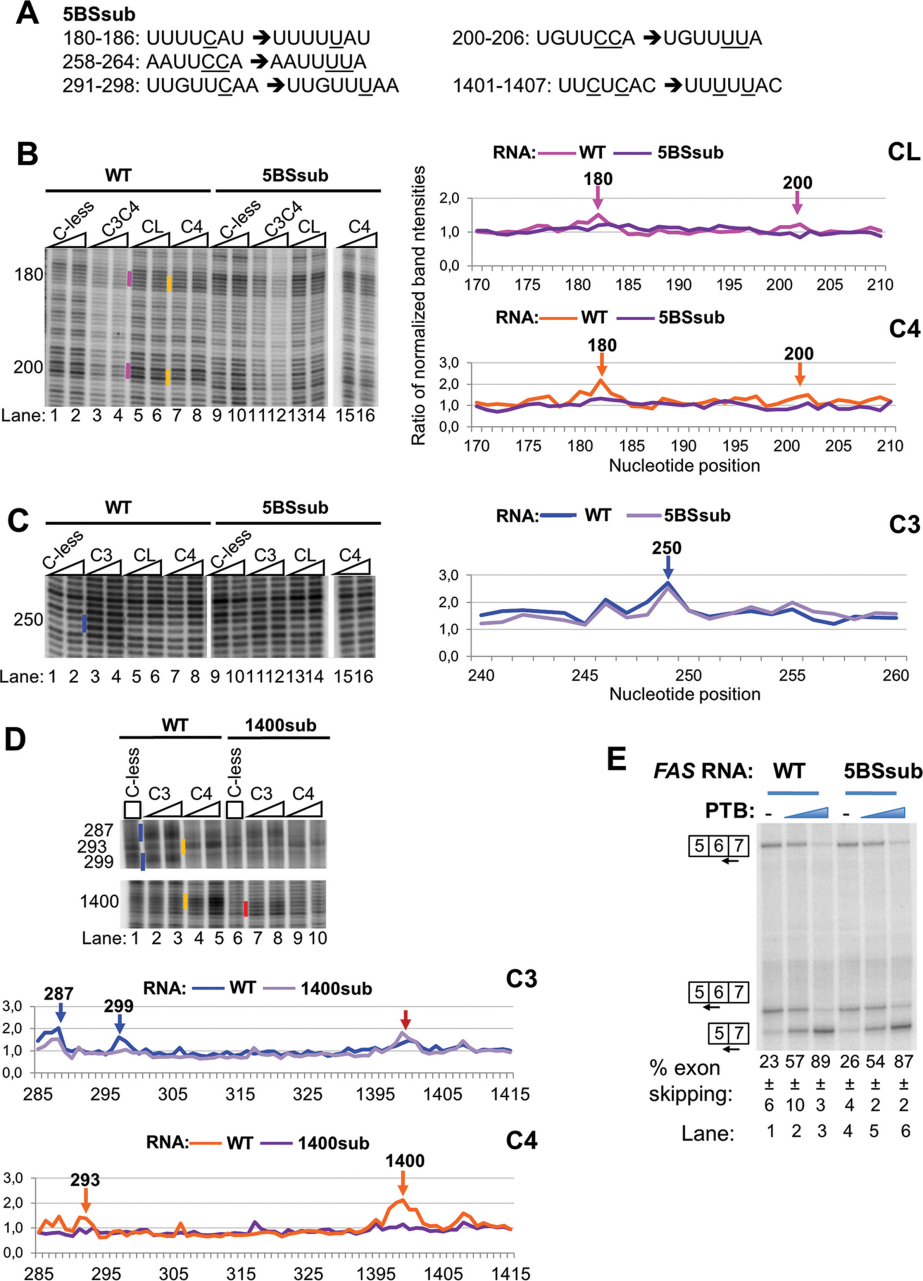
Effects of mutations of the RRM4 binding sites on the RNA upon PTB activity and binding. (A) Sequences showing the mutations introduced in the indicated nucleotide position of the *FAS* RNA to generate the 5BSsub mutant. The 1400sub mutant RNA had only the mutations shown in nts 291-298 and 1401-1407. (B) and (C) Fe(II)-BABE Cys PTB mutants were used in directed hydroxyl radical probing assays with *FAS* WT (lanes 1-8) or 5BSsub mutant RNA (lanes 9-16) and analysed as described in Figure 2A. Cleavage sites are indicated by vertical lines on the left of the corresponding bands on the gel, following the color-coding described in Figure 2. For clarity, only portions of the gels with the cleavage sites of interest are shown. Graphs derived from quantification of the gels using the SAFA software, as described for Figure 4 are presented. The peaks indicated by arrows correspond to the bands marked by vertical lines in the gels. (D) Fe(II)-BABE Cys PTB mutants were used in directed hydroxyl radical probing assays with *FAS* WT (lanes 1-5) or 1400sub mutant RNA (lanes 6-10) and analysed as described in Figure 2A. Graphs are shown as described for panels B and C. The red arrow indicates the stronger cleavage cut produced by C3 in the absence of RRM4 binding. (E) *FAS* WT (lanes 1-3) or 5BSsub mutant (lanes 4-6) RNA was spliced in vitro in HeLa nuclear extract in the absence (-) or presence of recombinant PTB, added to 10 and 30 ng/ml (blue wedges). Specific splicing products were detected by primer extension with end-labeled primers. The percent exon skipping shown below each lane is the average ± sd of three replicates.

The PTB mutant proteins were expressed, purified and derivatized as described by (15,18).

### Tethered hydroxyl radical probing

All *FAS* constructs were linearized with BstBI prior to transcription by T7 RNA polymerase, as described in (15). For tethered hydroxyl radical probing, 3.3 pmol of unlabeled RNA was heated briefly at 95°C and snap cooled after the addition of binding buffer (20 mM HEPES-KOH, pH7.4, 100 mM KCl and 2.5 mM MgCl_2_). Upon addition of the appropriate amount of Fe(II)-PTB mutant the complex was allowed to assemble for 30–45 min at 30°C, in a total volume of 33 μl with the presence of nuclear extract (9% v/v), 1 mM ATP and 20 mM creatine phosphate (CP). The reaction was then put on ice for 5–10 min, and 0.7 μl 250 mM freshly prepared ascorbic acid and 0.7 μl 1.25% H_2_O_2_ were added to initiate the Fenton reaction. After incubation for 10 min on ice, the reaction was stopped by adding a volume of stop solution (0.3 M NaOAc, 2% sodium dodecyl sulphate (SDS), 30% glycerol). The resulting RNA fragments were purified by phenol/chloroform extraction and ethanol precipitation and analysed by primer extension using AMV-RT and a series of ^32^P-5′end labeled DNA primers, scanning the whole sequence of *FAS* RNA, as described by (16). Usually, one-fifth of the re-isolated RNA was used for one primer reaction. The reverse transcription products were analysed by urea-acrylamide gel electrophoresis followed by phosphorimager analysis. Gels of different acrylamide concentrations and different running times were used in order to bring the cleavage bands at the best resolving area of each gel. The oligonucleotide sequences that were used for primer extension to probe the whole sequence of *FAS* RNA are F447R 5′ GTGCTTTCTGCATGTTTTCTG 3′; F366R 5′ GTAGAATGCATGTGAGAAC 3′; F289R 5′ GCAAGAACTTACCCCAAAC 3′; F212R 5′ GTAGGTTGGAACATTGGAC 3′; F136R 5′ GAGTGATTGGTTTTTCTTCAC 3′; F63R 5′ CCTTCCTCTTTGCACTTGG 3′.

### Quantification of the probing gels

To estimate the relative efficiency of cleavage at different sites, we used the Semi-Automated Foot-printing Analysis (SAFA) software (36,37), which facilitated a high-throughput quantification of the intensity of all bands on the primer extension gels, as described by (15). Briefly, to compensate for possible variations in loading, band intensities were normalized with respect to background bands, whose intensity was largely invariant in all lanes (including the Cys-less control lane). This normalized intensity for each individual band in a given lane was compared with the intensity of the corresponding normalized band in the Cys-less control lane. In all graphs presented in this study, the derived ratios were plotted against the nucleotide position in the *FAS* RNA. To generate the map of PTB-*FAS* RNA interactions presented in Figure 2C, the calculated ratios for all cleavage product bands were ranked, and the ranking list was divided into two groups (representing strong and weak), following common practice and similar to our previous analyses of PTB interactions with the EMCV and PV IRESs (15,16). If the ratio was 2 or greater it was classed as a strong cleavage, with weak cleavage assigned to ratios in the range 1.2–2. The map presented in Figure 2C is the outcome of such analyses of a large number of gels that were produced by primer extension analyses of a large number of probing reactions with different primers. It should be noted that some weak cleavage positions showed variable intensity among experiments.

### Filter binding assays

^32^P labelled-*FAS* RNA was allowed to form a complex with recombinant PTB. Usually, RNA was used at a final concentration of 2–4 nM, with the protein ranging between 1 and 2200 nM. The complex was assembled at room temperature for 15–30 min in 5 mM Hepes pH 7.4, 100 mM KCl, 3 mM MgCl_2_, 5% glycerol, 75 μg/ml yeast tRNA, 75 μg/ml BSA and 0.5 mM DTT, at a final volume of 50 μl. Assays were performed using the protein-binding Protran NBA-085B nitrocellulose membrane (Whatmann) to immobilize the PTB–RNA complexes and a lower layer of Hybond-N membrane (Amersham Life Sciences) to immobilize the unbound free RNA. The membranes were washed extensively in 10 mM HEPES pH 7.4, 2.5 mM MgCl_2_, 5% glycerol, 1 mM DTT and mounted on a 96-well dot-blotter. Before and after application of 45 ml of the binding reaction, the membrane was washed with 180 ml of wash buffer. Following the experiment, the membrane was dried, and the fraction of input RNA bound to PTB was determined by phosphorimager and analysis using TotaLab TL120 software (Nonlinear Dynamics). To calculate the apparent Kd values for each PTB mutant, the ratio of bound/free RNA was plotted against protein concentration. Kd values were determined from the slope of the straight lines generated from these graphs.

### *In vitro* splicing

100 fmol of capped *FAS* RNA was incubated with HeLa nuclear extract (20% v/v) in the presence of 2.2 mM MgCl_2_, 1 mM ATP, 20 mM CP, 12U RNasin and 88 mM KCl in a total volume of 10 μl. After incubation at 30°C for 30 min, the reaction was stopped by the addition of 20 μg proteinase K and incubation for a further 30 min in a buffer containing 100 mM Tris pH 7.5, 12.5 mM EDTA, 150 mM NaCl and 1% SDS. After phenol extraction and ethanol precipitation, the RNA products were used for primer extension reactions. Usually, one splicing reaction would be enough for four primer extension experiments. The oligonucleotides used for primer extension analysis were: EJ5-7: 5′ CCTTTCTCTTCACTTCCTC 3′; EJ5-6: 5′ CAAGTTAGATCTGGATCCTTCC 3′; EJ6-7: 5′ TCCTTTCTCTTCACCCAA 3′.

### Total internal reflection fluorescence (TIRF) analysis

Single molecule experiments were carried out as previously described (25,26) with minor modifications. For RNA labelling an oligonucleotide ATTO 647N-5′-TUGUCUCCCAU-3′-biotin complementary to the first nine nucleotides of RNA was used (Eurogentec, Belgium). Underlined bases are LNA; all others are 2′-*O*-Me. To diminish the amount of non-specifically adsorbed PTB-GFP molecules due to its high concentration (around 10 μM) in overxpressed nuclear extract (a gift from C. Gooding), the latter was diluted 10- to 20-fold with unlabelled nuclear extract. Complexes were formed by incubating labelled RNA at 100 nM with the diluted nuclear extract (50%, 10 μl incubation volume) at 30ºC for 30 min (25,26). The incubation mixture was diluted in 10 mM Hepes-HCl, pH. 7.5, 50 mM NaCl to a final RNA concentration around 1–5 pM and 25 μl of this mixture was injected into the microscope chamber. Note that 3–5 min were required to settle the complexes onto imaging surface of silica slide. Prior to TIRF experiments silica slides were PEG-functionalized (38) and activated with Neutravidin (Invitrogen). Acquisitions were recorded for 500 time bins (200 ms/bin) starting with ATTO 647N (633 nm laser excitation) for 30 bins with the rest for GFP (488 nm laser excitation) using house-built dual imaging system (525/40 emission filter, Semrock; 540DCLP dichroic mirror, Omega and 670 DF40 emission filter, Omega) projecting split signals from each colour on the corresponding half of the emCCD (iXon DV887, Andor) chip. 20–25 acquisitions were collected for each experiment.

Time-series intensities for co-localized spots were extracted from 10 × 10 pixel areas and background corrected. For each experiment a distribution of the number of bleaching steps together with a cumulative distribution of collected emissions were built. In addition, a cumulative distribution of collected emissions was built for the complexes showing one-step behaviour only. To calculate the number of PTB target sites a knowledge about fractional occupancy of PTB target sites by PTB-GFP molecules, *θ*, at the conditions used for complex formation is required. The latter depends on the number of target sites, total PTB and RNA concentrations and PTB and PTB-GFP affinity constants in a non-linear manner. Usage of cross-linking data extracted from the experiments carried out at very low RNA concentration around 1 nM might lead to an underestimation of PTB-GFP bound fraction at RNA concentration of 100 nM by 15%–30% at low molar protein/RNA ratio (3–5 in our case) and high number of target sites (3–6 in our case). To overcome these uncertainties we used following procedures for evaluation of the number of specific sites.

To estimate the number of target sites from the distribution of bleaching steps, a distribution was fit by the truncated binomial distribution for various number of the specific sites, *n* = 2, 3… 8, by minimizing *χ*^2^. The *n* value that gave the minimum for *χ*^2^ was taken as a good approximation for the number of specific sites. Alternatively, we plot values *iP_i_*/*P*_(*i*−1)_ versus *i*, where (*i*) is the number of bound PTB-GFP molecules, *P_i_* and *P*_(*i*−1)_ are the occurrences for the complexes with (*i*) and (*i*−1) bound molecules determined from the distribution of bleaching steps. Approximation of data by a straight line gave estimates for *n* and *θ* (39). To evaluate the number of target sites from the cumulative distribution of emitted intensities, we first fit cumulative distribution for the complexes showing singular bleaching behaviour by the cumulative distribution of exponential function that gave us estimate for the average value of emitted intensities of GFP molecules (25,26). This allowed fitting cumulative distribution for the entire set by the corresponding cumulative function using estimate for *θ* given by the analysis of bleaching steps. To verify our analysis we used *Tpm1* RNA for which the number of PTB target sites was determined as 5–6 (26). We note that uncertainty in estimation of *n* by either approach is close to one. The data shown in Table 2 represent typical values drawn from several experiments for each RNA tested.

**Table 1.** Apparent affinity of PTB RRM mutants for *FAS* RNAs

RNA	Protein	K_D_ (nM)
WT *FAS*	C1C2	10.1 ± 3.8
	C1_m_C2	11.6 ± 3.9
	C1C2_m_	15.3 ± 6.7
	C1_m_C2_m_	12.3 ± 1.7
	C3C4	14.7 ± 0.9
	C3_m_C4	16.5 ± 2.3
	C3C4_m_	22.9 ± 6.5
	C3_m_C4_m_	24.6 ± 4.3
	C4	10.3 ± 2.6
	C4_m_	29.9 ± 5.6
	C4pack	6.3 ± 2.3
	L_m_C4	14.2 ± 2.2
m0 mutant	C4	7.5 ± 1.6

**Table 2.** Single molecule analysis of PTB target sites on *FAS* RNA

RNA	Number of PTB target sites			Fraction of RNA with bound PTB-GFP, %
	A	B	C	
wt *FAS*	3–4	3–4.5	3–4	2.6
m0 mutant	3	2–4	3	2.9
*Tpm* 1 RNA	5–6	6.5	6	7.9

(A) the number of the target sites was estimated by fitting distribution of bleaching steps by the truncated binomial distribution; (B) the number of the target sites was estimated by linear fit of *iPi*/*P*(*i*−1) values; (C) the number of the target sites was estimated from the analysis of total emissions (see Materials and Methods). The data in the last column were obtained at 1:20 dilution of PTB-GFP overexpressesing nuclear extract.

## RESULTS

### Differential effects of PTB RRM mutations

The tethered hydroxyl radical probing is based on a ‘Cys-less’ parental PTB1, lacking amino acids 1–55, and with cysteines 250 and 251 in RRM2 mutated to serine (Figure 1A) (15). Individual cysteines are introduced at locations close to the RNA binding surface of each RRM, taking care to minimize effects on PTB function (Figure 1A, Supplementary Figure S1). Modification of cysteine with the Fe(II)-BABE reagent then allows interrogation of RNA contacts by the associated RRMs (15). The resulting PTB constructs are named C1, C2, C3, C4, C1C2, C3C4 indicating the RRMs in which the cysteines are located; CL refers to the N432C mutant with a cysteine in the linker between RRM3 and 4 (Figure 1A). In later experiments the single cysteine mutations were combined with mutations to impair RNA binding by individual RRMs (Figure 1B, Supplementary Figure S1). The *FAS* exon 5–6-7 construct with a 1060 nt deletion in the intron between exons 6 and 7 (subsequently referred to as WT *FAS*) was spliced in HeLa cell nuclear extract, and predominantly included exon 6 (22% exon skipping, Figure 1D, lane 1), as previously reported (29). Addition of recombinant PTBs (Figure 1C) with one or two cysteine substitutions produced a dose-dependent increase in exon skipping, with ∼90% exon skipping upon addition of 30 ng/μl (∼0.5 μM) PTB (Figure 1D, lanes 2, 3, 10, 11, 15, 16, 20, 21, 25, 26). This activity is comparable with unmodified PTB, which also caused maximal exon skipping at the same concentration (data not shown), consistent with the design of these mutants to facilitate monitoring of protein-RNA interactions while minimally affecting PTB function (15). We next tested the sensitivity of PTB splicing repressor activity to mutations designed to impair RNA binding by individual RRMs (18) (Figure 1B). The RNA binding mutations were introduced into single or double cysteine mutants, with one cysteine always in the inactivated RRM. This allowed us to monitor the effect of each RRM mutation upon PTB-RNA contacts using tethered hydroxyl radical probing (see below). Mutations to impair RNA binding are indicated by the subscript ‘m’ after the mutated RRM. For example, C3C4_m_ has cysteines introduced in RRMs 3 and 4, and RNA binding mutations in RRM4; tethered hydroxyl radical probing with C3C4_m_ could show us whether the mutation inactivating the RRM4 has the expected effect in RRM4-RNA contacts and whether it also affects RRM3-RNA contacts (see below).

All of the single RRM binding mutants showed reduced repressor activity compared to their parental single- or double-cysteine PTB. However, the effects of the RNA binding mutations varied widely. The RRM2 mutant (C1C2_m_) was least impaired (Figure 1D, lanes 7–9; Figure 1E) with 80% of WT activity at 30 ng/μl, followed by the RRM1 (C1mC2) mutant with 54% of WT activity (Figure 1D, lanes 4–6; Figure 1E, see legend for calculation of % activity). The mutations in RRM3 and RRM4 were more disruptive, reducing activity to 38% and 19% of WT, respectively (Figure 1D, C3_m_C4, lanes 12–14, C4_m_, lanes 17–19; Figure 1E). The observation that the RRM4 mutation was most deleterious to function is striking given that RRM4 has the lowest inherent binding specificity of all four RRMs, recognising only a YC dinucleotide (11). We next tested the effects of double mutations in RRMs 1 and 2 and RRMs 3 and 4. The RRM1-2 double mutant (C1_m_C2_m_) was more impaired than the RRM2 single mutant, but slightly more active than the RRM1 single mutant, with 65% activity at 30 ng/μl (Figure 1D, lanes 27–29; Figure 1E). In contrast, the RRM3-4 double mutant (C3_m_C4_m_) was less active than all other mutants, with only 16% of WT activity (Figure 1D, lanes 22–24; Figure 1E). Increasing the concentration of C1_m_C2_m_ mutant 2-fold (to 60 ng/μl) substantially rescued activity (86% of WT activity, Figure 1D lane 29), but had a more modest effect for C3_m_C4_m_ (34% of WT activity, Figure 1D, lane 24). Filter binding assays indicated that all mutants showed only modest reductions in affinity for *FAS* RNA, with apparent dissociation constants increasing by at most 3-fold (Table 1), similar to the effects upon binding to EMCV IRES (18). Notably, RNA binding by RRM12 and RRM34 double mutants is specific; mutation of all four RRMs leads to a drastic decrease in affinity of more than 2000-fold (C. Gooding and CWJS, unpublished observation). Moreover, the concentrations of PTB in the splicing assay are 20- to 50-fold higher than the determined K_d_s. Thus, the effects of the RRM mutants upon splicing are unlikely to be explained by decreased affinity for RNA, but rather by the disrupted architecture of RRM-RNA interactions. Furthermore, these results indicate that RNA binding by the RRM3-4 di-domain appears to have a more central role in splicing repression than the N-terminal RRMs 1 and 2.

### Tethered hydroxyl radical probing of PTB RRM interactions with *FAS* pre-mRNA

We next used the single and double cysteine PTB mutants to generate a map of the interactions of the PTB RRMs with the WT *FAS* pre-mRNA (Figure 2). All single Cys and double mutants were active as splicing repressors (Figure 1D, lanes 2, 3, 10, 11, 15, 16, 20, 21, 25, 26). Tethered hydroxyl radical probing was carried out using PTBs with single Cys residues at the positions indicated in Figure 1A: Q124C (C1 in RRM1), D139C (C1A), D242C (C2A), D284C (C2 in RRM2), N395C (C3 in RRM3), N432C (CL in the linker between RRM3 and 4), E518C (C4 in RRM4). Sites of cutting were assessed by primer extension. Probing was assessed by comparison with a ‘dummy’ reaction with the ‘Cys-less’ PTB control, which provides a background level of RNA cleavage and reverse transcriptase stops (Figure 2A and B). Experiments were initially carried out with pure protein and RNA, as previously (15,16), and subsequently in the presence of HeLa nuclear extract under similar conditions to the splicing assays (Figure 2A and B). Similar patterns of RNA cleavage were observed under both conditions, but the signal-to-noise ratio was significantly better in the presence of nuclear extract, most likely due to reduction in non-specific binding due to the presence of other RBPs. All subsequent probing experiments were therefore carried out in nuclear extract.

Examples of probing gels are shown in Figure 2A and B, and a summary of all observed interactions is shown in Figure 2C. Note that the positions of the cuts are numbered according to the sequence of the *FAS* RNA with no deletion between exons 6 and 7, whereas all experiments shown used the construct with a 1060 nt deletion; consequently, position 327 is immediately adjacent to position 1388. A number of features are evident from these probing data. First, all RRMs show multiple points of contact with the *FAS* pre-mRNA. The strongest contacts were observed for RRM3, the RRM3-4 linker and RRM4. Consistently, the most prominent cuts were produced by probing from RRM3 (C3), indicating strong contacts at position ∼248–250 (Figure 2A compare lane 7 with 13), within the previously identified PTB-binding silencer in exon 6 (URE6, (29)), and at ∼287–289 just downstream of exon 6 (Figure 2A, lane 7 compare to C-less lane 13; Figure 2B lane 7, compare to C-less, lane 12). The latter location corresponds to an element called URI6 (U-rich intron 6), which mediates activation of exon 6 by TIA-1, but is not required for repression by PTB (29). Cuts produced by probing from RRM4 (C4) and the RRM3-4 linker (CL) were all co-located, within 1–2 nt of each other. This could be because the CL and C4 constructs both cut at a segment of RNA that links the sequences bound to the two RRM β-sheet surfaces, which would be roughly equidistant between N432C and E518C (see below, Figure 1, Supplementary Figure S1). The strongest interactions were observed at positions ∼180, 293 and 1400 (Figure 2A, lanes 9, 11; Figure 2B, lanes 9, 11). Additional weaker contacts were observed at 73–74, 200 and 259. In five of the six cases, the RRM4 cuts were within 2 nt of a downstream UC dinucleotide (Figure 2C), consistent with the YC preference observed in the NMR structure of RRM4 bound to CUCUCU RNA (11). Probing with RRM1 single Cys PTB showed six weak sites of contact. RRM2 showed only 3 weak contact points, with diffuse rather than sharp bands, possibly indicating a looser contact of RRM2 with the RNA. The relative strength of contacts by RRMs 1 and 2 compared to RRMs 3 and 4 are consistent with the more detrimental effects of RRM 3 and 4 RNA binding mutations upon PTB splicing repressor activity (Figure 1).

The strong interactions of RRM3 at the URE6 location are consistent with the exon splicing silencer activity of this element (29). In addition, weaker cuts from RRMs 1, 2, 4 and the RRM3-4 linker were also observed around the URE6. The RRM4 and linker cuts were ∼10 nt downstream of the strong RRM3 cuts, consistent with the smallest possible loop size between the RNA contacts at RRM3 and 4 (11,13). This suggests that a single PTB molecule binds at the URE6 location. However, the weakness of the RRM4 and linker cuts at 259, coupled with the excess of RRM4/linker contacts suggests that the RRM4 of the PTB bound at the URE6 can interact at more than one location on the *FAS* RNA. The observation of RRM contacts at numerous other locations was unexpected, and could be explained by multiple PTB binding events to individual RNAs. Alternatively, it is possible that a single PTB binds to *FAS* RNA in multiple alternative configurations. To distinguish between these possibilities we used TIRF microscopy (26) to examine the binding of GFP-PTB in nuclear extracts to *FAS* RNA. The results of TIRF experiments showed that 3–4 PTB molecules could bind WT *FAS* RNA (Table 2). This was indicated by analysis of distributions of bleaching steps and emitted intensities (25,26) (see Materials and Methods). Furthermore, comparison with *Tpm*1 RNA, for which the number of PTB target sites was determined earlier (26) and confirmed here, indicated that *FAS* RNA has fewer PTB binding events than *Tpm*1 RNA. Our estimates indicate that an average dissociation constant for PTB binding with *FAS* RNA is around 20 nM (not shown), consistent with the filter binding assays (Table 1). Previously, titration of Fe(II)-BABE-modified PTB into probing reactions with EMCV IRES showed separate groups of modifications that appeared at different protein concentrations (15), reflecting two binding events with substantially different affinities. We attempted the same analysis with *FAS* RNA, but found that most cuts appeared over a similar PTB concentration range, suggesting that the multiple binding events have similar apparent affinities (data not shown).

We next analyzed the effects of RNA binding mutations upon RNA contacts by the different RRMs (Figure 3). In all cases, mutations designed to impair RNA binding abolished all observed contacts by that RRM. For example, the strong contact of RRM3 with the URE6 at position 250 was abolished by mutation of RRM3, but was unaffected by mutation of RRM4 (Figure 3A, lanes 4, 5, 6, 7 compared to lane 2). Combined with the detrimental effect of RRM3 mutation upon activity (Figure 1), this suggests that the interaction of RRM3 with the URE6 is necessary but not sufficient for PTB repressor activity. Likewise, the contact of RRM4 at 180 (Figure 3A, lane 3) was abolished by RRM4 mutation (lanes 6, 7), but unaffected by RRM3 mutation (lanes 4, 5). In most cases, specific RNA contacts were only affected by mutations in the same RRM (Figure 3, and Supplementary Figure S2 for RRMs 1 and 2). The exception was the contact of RRM 3 at positions ∼287 and 299 just downstream of exon 6, which was significantly reduced by mutations in RRM4 as well as in RRM3 (Figure 3B, lanes 8–13 compared to lanes 2–5). This suggests that at this site the interaction of RRM3 is stabilized by interactions of RRM4 with RNA. The fact that individual RRM mutations only affected contacts by that RRM is consistent with their relatively modest effects on affinity (Table 1).

### Effects of RNA mutations upon PTB RRM contacts and splicing activity

Having mapped the locations of PTB RRM contacts with *FAS* RNA, we next tested the importance of some of these sites, with a focus on RRM3 and 4 contacts since these RRMs are functionally more important (Figure 1). The strongest contacts were observed between RRM3 and the previously identified URE6 silencer. As previously reported, mutation of URE6 (m0 mutant Figure 4A, (29)) reduced the level of exon skipping in nuclear extract (Figure 4B lanes 1 and 4) and severely impaired the ability of PTB to induce exon skipping (Figure 4B, lanes 1–6). When the m0 mutant was compared with WT RNA in tethered probing experiments we found that all RRM-RNA contacts within URE6 were affected (Figure 4C). In particular, the strong RRM3 and RRM1 contacts were abolished (Figure 4C, lanes 2, 3, 6, 7, 14, 15, 18, 19), as was the weaker contact of RRM2 (lanes 4, 5, 16, 17). All other RRM-RNA contacts outside of URE6 remained intact, with the possible exception of the weak contacts of RRM4 and the linker at 259, just downstream of URE6. In some probing gels (although not clearly evident in Figure 4) these contacts were reduced, suggesting that this RRM4 contact is associated with the strong RRM3 contact within URE6. Filter binding assays indicated no significant effect of the m0 mutation upon apparent affinity for PTB (Table 1). This was supported by the unchanged fraction of RNA co-localizing with GFP-PTB in single molecule assays (Table 2). However, m0 was observed to bind fewer PTB molecules than WT *FAS* (Table 2). The reduction in number of bound PTBs was not sufficiently pronounced to allow a clear-cut conclusion that URE6 deletion leads to loss of one binding event, as might be expected. A possible explanation for these observations is that altered global RNP organization in response to URE6 deletion allows an alternative PTB binding event on a fraction of *FAS* RNAs possibly at more than one location (see Discussion). The suggested heterogeneity of complexes would be consistent with the lack of new contacts observed by tethered probing of the m0 mutant (Figure 4C).

We next examined the effects of a C to U transition mutation at position 285 in the URI6 region, which disrupts a UUCUU motif just upstream of the strong RRM3 contact at 287 (Figure 4A, URI6m). This mutation led to a substantial reduction in RRM3 cuts at 287, but not at 250 (Figure 4D, lanes 11, 12 compared to 3, 4, Figure 4E). RRM4 and linker cuts at position 293 (which were weak in this experiment) were also reduced, but were unaffected at position 1400 (Figure 4E, middle and lower panels). In contrast, the URI6 mutation had no effect upon the ability of PTB to repress splicing of *FAS* exon 6 (Figure 4B, lanes 7–12), in agreement with the maintained PTB-responsiveness upon a more severe mutation at this site (29). The preceding data are therefore consistent with the idea that the PTB RRM3 contacts with *FAS* pre-mRNA at the URE6 location are key for its repressive effects.

The RNA binding mutations within the PTB RRMs showed that impairment of RRM4 binding had the severest effect upon splicing repressor activity (Figure 1D and E), despite RRM4 having the lowest intrinsic binding specificity, recognizing just a YC dinucleotide within a CUCUCU hexamer (11). We noted that at 5 of 6 contact points of RRM4 with *FAS* RNA, the cuts were ∼1–2 nt upstream of a UC dinucleotide (Figure 2C). To test, whether these YC dinucleotides were necessary for RRM4 contacts, and for PTB repressor activity, we mutated them to UU dinucleotides by one or two C to U transitions (Figure 5A). Examination of cutting at these locations indicated that contacts by RRM4 and the linker (C4 and CL probes) were substantially reduced or abolished at positions 180, 293 and 1400 (Figure 5B and D). Unexpectedly, the reduced contacts by RRM4 at 1400, were accompanied by increased contacts by RRM3 (Figure 5D). In contrast, other contacts, such as that of RRM3 at 250, were unaffected, whereas the 287 and 299 contacts by RRM3 were reduced (Figure 5C and D). In these experiments the weaker RRM4 contacts at 200 and 259 were not readily observable with WT *FAS* RNA, so we could not discern the effects of the C to U transitions at these locations. Nevertheless, it is striking that at the locations where RRM4 and linker contacts were most readily observed with WT RNA, they were abolished by conservative pyrimidine transitions. We examined splicing of the mutant *FAS* carrying C to U mutations at all five positions (5BSsub). Splicing of WT *FAS* and 5BSsub was indistinguishable, in the presence or absence of added PTB (Figure 5E), suggesting that none of the sites of RRM4 contact are critical for PTB repressor activity. A possible explanation for the discrepancy between the severe effects of mutations in RRM4 upon PTB repressor activity (Figure 1D and E) and the lack of effect of mutations at its preferred RNA contact sites (Figure 5E) is that RRM4 can readily find alternative, functionally redundant, YC contact points on the RNA. We examined the probing gels of the 5BSsub RNA but were unable to detect the appearance of any major new RRM4 contacts. It is possible that upon mutation of the preferred sites, multiple alternative RRM4 contact sites can be used, with insufficient signal-to-noise at any individual site to allow their detection (see Discussion). Another possible explanation for the lack of functional effect might be that enhanced RRM3 contacts at 1400 compensate for reduced RRM4 contacts. If this were the case, we would expect the 5BSsub RNA to show enhanced sensitivity to the RRM3 binding mutation, and reduced sensitivity to the RRM4 binding mutation. However, we found that 5BSsub was indistinguishable from WT *FAS* in its response to wild-type PTB and the RRM3 and 4 binding mutants (data not shown).

### Importance of the RRM3-4 di-domain

**Figure 6.**
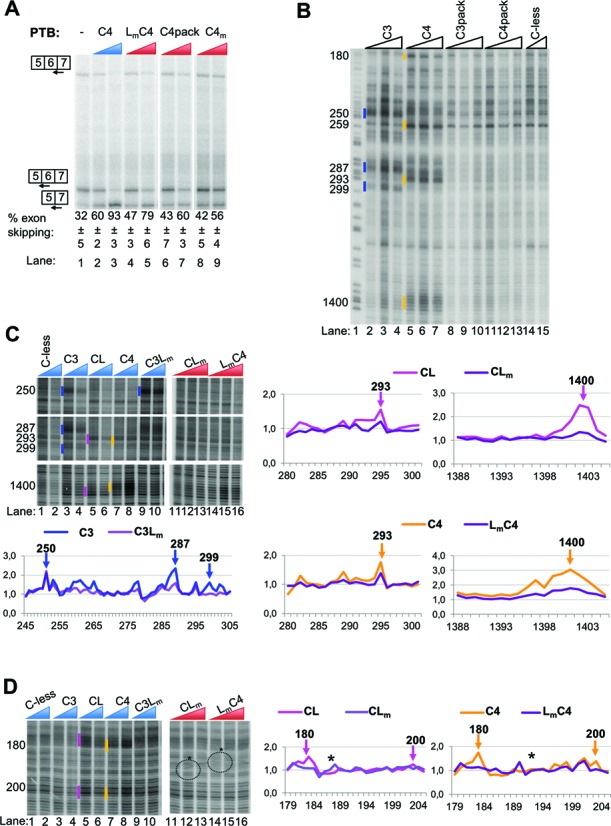
Effects of mutations altering the PTB RRM3-4 di-domain on its splicing and RNA-binding functions. (A) *FAS* WT RNA was spliced *in vitro* in HeLa nuclear extract in the absence (−) or presence of recombinant PTB mutants as indicated. PTB mutants were added to 10 and 30 ng/μl. Specific splicing products were detected by primer extension with end-labeled primers. The percent exon skipping shown below each lane is the average ± SD of three replicates. (B) Fe(II)-BABE Cys PTB mutants were used in directed hydroxyl radical probing assays. 0.1 μM of *FAS* WT RNA were incubated with 0.9, 1.2 μM or 1.5 μM Fe(II)-BABE-PTB mutants. Analysis was performed as described in Figure 2B. Cleavage sites are indicated by vertical lines on the left of the corresponding bands on the gel. Cuts produced by the lower amount of derivatized protein added are indicated. The colour-coding introduced in Figure 2 is also applied here to depict cleavages produced by cysteines in the different RRMs of PTB. Lane 1 (*T*) depicts a sequencing ladder generated by the same primer. (C) and (D) Fe(II)-BABE Cys PTB mutants were used in directed hydroxyl radical probing assays as described above. 0.1 μM of *FAS* WT RNA were incubated with 0.9 and 1.2 μM (blue wedges) or 0.9, 1.2 μM and 1.5 μM (red wedges) of Fe(II)-BABE-PTB mutants. Analysis was performed as described above. Cleavage sites are indicated by vertical lines on the left of the corresponding bands on the gel, as described for panel B. The graphs presented within each panel have been produced from quantification of the relevant gel by SAFA software. Peaks corresponding to the bands indicated by vertical lines in the gels are shown by arrows. The exact nucleotide positions of the cleavage sites are indicated by the numbers above the arrows. The asterisks at the gel and the graphs in panel D show artefact-signals produced during the processing of the gel.

The preceding data indicated the importance of RNA contacts by RRM3 and 4 for PTB repressor activity, despite the low specificity of RRM4 contacts. RRM3 and 4 form a stable di-domain with back-to-back packing of the two RRMs involving the highly conserved linker (11,40). Mutation of the packing interface decreased affinity of recombinant RRM34 di-domain for RNA, and reduced repressor activity of full length PTB upon the *CSRC* N1 exon in a co-transfection assay (13). We therefore decided to test the packing mutant, C4pack (E502K; V505E; I509K, all in helix 2 of RRM4) for its effects upon *FAS* splicing (Figure 6A). The packing mutant showed a substantial reduction in repressor activity, with 46% of WT activity at 30 ng/μl (Figure 6A lanes 6, 7), while the RRM4 binding mutant had 39% of WT activity in this experiment (Figure 6A, lanes 8, 9). We tested the effects of the packing mutation upon RNA contacts by RRMs 3 and 4 by tethered OH radical probing. All contacts by RRMs 3 and 4 were abolished by the packing mutant (Figure 6B), indicating that stable RNA contacts by both RRMs are dependent upon their back-to-back packing. In contrast, no deleterious effects of the packing mutation were observed in a filter binding assay (Table 1).

A possible role for the di-domain packing is the organization of a high density of surface positive charge in the properly packed di-domain (41). This positive patch might provide a binding surface for the ‘looped’ RNA between the specific motifs recognized by RRMs 3 and 4 (13,41). Indeed, chemical shift changes in this region were observed when RRM34 di-domain was bound to RNA with two pyrimidine tracts separated by a run of A's (13). The positive patch is formed by residues in the RRM3-4 linker (H436, R437, K439, K440) and in RRM4 (H457A, β1 strand; K485A, β2 strand). H457 and K485 are involved directly in RNA binding by RRM4 (11,42), while H436 and R437 are conserved in nPTB, and in the crystal structure of the nPTB RRM3-4 play a role in di-domain packing (43). In contrast, the linker residues K439 and K440 are solvent exposed and conserved across PTB and its paralogs (44), but do not play a role in di-domain packing (Figure 6, Supplementary Figures S1 and S3). We therefore created a K439/440A double mutant (linker mutant, L_m_) to test the role of the di-domain positive patch. The L_m_ mutant had activity upon *FAS* splicing intermediate between that of wild-type PTB and the packing mutant, with 77% of the WT activity compared to 46% for the packing mutant (Figure 6A, lanes 4, 5). To test the effect of the L_m_ mutant upon PTB-RNA contacts, we combined it with the C3, CL and C4 single cysteine mutants for tethered hydroxyl radical probing experiments (Figure 6C and D). The K439/440 residues targeted in the L_m_ mutant are 9.4 and 12 Å from the N432C substitution site to probe linker-RNA interactions. Consistent with this, all RNA contacts detected by the N432C probe (CL) were abolished by the K439/440 mutation (Figure 6C and D). This effect was most obvious with the strongest cuts at the 1400 location (Figure 6C), but it was also evident at other contacts detected by N432C. Likewise, all RNA contacts by RRM4 were strongly reduced by the L_m_ mutation, with the most obvious effects at the strong 1400 contacts (Figure 6C and D). This could either be because binding at the β-sheet surface of RRM4 is stabilized by the linker interactions or because E518C is sufficiently close to K439/440 to produce cuts directly on RNA bound at the linker region.

RRM3 contacts at the URE6 location (position ∼250) were unaffected by the linker mutation, but were reduced at position 287, 299 (Figure 6C). This is consistent with the fact that RRM3 contacts at 287 and 299, but not at 250, were affected by RRM4 mutations (C4m, Figure 3B), and suggests that the linker residues K439/440 contribute directly to the stability of RRM4-RNA interactions, effectively providing a more extended RNA-binding surface for RRM4. RRM3 contacts with RNA are therefore only affected by the linker mutation when they are dependent upon RRM4-RNA contacts.

The preceding data suggest that at least part of the role of the di-domain packing is to create a positive surface patch to which RNA between the motifs recognized by RRMs 3 and 4 can bind. This patch of surface positive charge effectively extends the binding surface provided by RRM4.

## DISCUSSION

Our data provide interesting insights into the way in which PTB interacts with a target alternatively spliced pre-mRNA substrate. Tethered probing showed the major set of contacts were within the previously characterized URE6 silencer (29) within exon 6, where RRM3 made the most prominent contact (Figure 2). Unexpectedly, numerous additional contacts from PTB RRMs were observed in both flanking introns. Consistent with this, single molecule analyses indicated that WT *FAS* pre-mRNA can accommodate 3–4 PTB molecules (Table 2). Other than the inhibitory PTB bound at the URE6, it appears that a second, non-inhibitory, PTB molecule binds at the URI6 location just downstream of exon 6 (Figures 2 and 4, (29)). The RRM3 contact at 287 is flanked by weak cuts from RRMs 1 and 2, with additional RRM4 and linker cuts only 6 nt downstream. Since a loop of at least 12 nt is needed between motifs bound to RRMs 3 and 4 (11,13), at first sight the adjacent RRM3 and RRM4 cuts at 287 and 293 appear too close to be associated with the same PTB molecule. However, the C4 probe (E518C) is suitably positioned to probe RNA bound either at the β–sheet surface of RRM4 or in contact with the cluster of positively charged linker side-chains (Figure 1, Supplementary Figures S1 and S3). Indeed, the fact that all linker and RRM4 cuts are immediately adjacent (Figure 2) suggests that the C4 probe may preferentially detect linker-bound RNA. The adjacent RRM3, linker and RRM4 contacts at 287–293 might therefore be associated with the same PTB molecule. Supporting this interpretation, the position 285 C to U mutation (URI6m) reduced not only RRM3 contacts at 287 (URI6m, Figure 4) but also linker and RRM4 cuts at 293 (Figure 4D and E). Other lines of evidence also indicated the interdependence of RRM3 and 4 contacts at this location; RRM3 cuts at 287 were reduced in response either to mutations inactivating RRM4 (Figure 3), or to mutations of the RNA contact points of RRM4 (Figure 5). This mutual dependency for binding was not observed at other locations, including the inhibitory URE6 location. The location of the additional PTB binding events indicated by single molecule analysis is less clear. One additional PTB might be associated with the strong RRM4 and linker contacts at 1400 and the RRM3 cut at 299 and there are a number of weak contacts from all RRMs in intron 5, so another PTB molecule might bind upstream of exon 6 (Figure 2). The difficulty in placing 3–4 PTB molecules could be related to the fact that not all *FAS* RNA molecules have the same complement of PTBs bound at identical positions. Chemical probing shows the sum of all interactions across all bound RNA molecules. Strong contacts, such as the functionally important interaction of RRM3 at URE6, might occur on a high proportion of RNAs, while weaker cuts might correspond to interactions that occur on a subpopulation of RNAs. For example, the three RRM1 cuts in URE6 (Figure 2) likely represent three possible conformations of binding in conjunction with the major RRM3 interaction.

**Figure 7.**
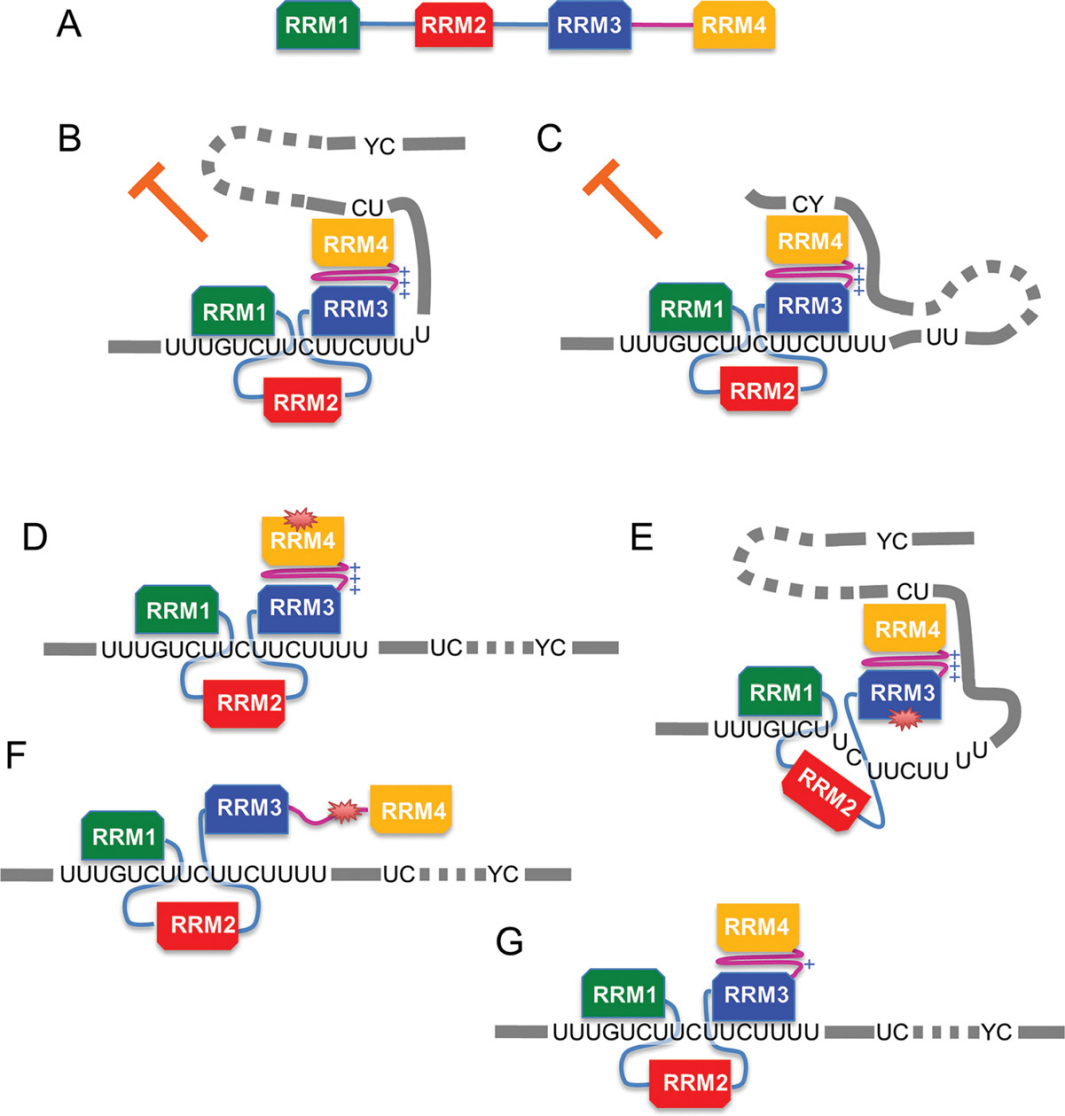
PTB repression of FAS exon 6 requires di-domain RNA contacts by RRMs 3 and 4. (A) Linear representation of PTB with RRMs and the RRM3-4 linker colour coded as in previous figures. RRMs are depicted with the longest side representing the RNA-binding β-sheet face. In panels B–G RRM1 and 3 are depicted binding at the URE6. (B) PTB is inhibitory when RRM3 binds at URE6 and RRM4 binds at a downstream YC dinucleotide, even when the proximal UC is mutated (C). PTB is not inhibitory when either RRM4 (D) or RRM3 (E) have an RNA binding mutation or when RRM3-4 packing is disrupted (F). Inhibitory activity is reduced when the surface positive charge organized by di-domain packing is reduced (G).

A related insight is that PTB binding events are not necessarily characterized by a 5′ to 3′ arrangement of contacts by RRMs 1, 2, 3 and 4, respectively. While the major sites of RRM3 contact at 250 and 287 are preceded by RRM1 and followed by RRM4 contacts, there is no intervening RRM2 contact. Indeed, in both cases a weak RRM2 contact lies just downstream of the RRM3 site. Other observations also suggest a high degree of flexibility in the arrangement of RRM-RNA contacts. Compared to the small number of strong contacts by RRM3, both RRMs 1 and 4 have a larger number of weaker contacts. For example, the RRM4 contact at 259, just downstream of URE6 was weak and, being located in an area with a high background of RT stops, was observed with difficulty in some experiments. This suggests that the RRM4 of the PTB associated with the URE6 has the flexibility to contact RNA at one of several locations, consistent with its low specificity for a YC dinucleotide (11) (Figures 5 and 7). An alternative explanation for the excess of RRM4 over RRM3 contacts is that some PTB molecules can bind *FAS* RNA without using their RRM3 domain. Indeed, mutation to impair RRM3-RNA interaction had no effect on apparent affinity for *FAS* RNA (Table 1), or most RRM4 contacts (Figure 3), although it significantly impaired activity (Figure 1D and E), so any binding events not involving RRM3 are unlikely to be functional.

Our data clearly indicated that for regulation of *FAS* splicing the various RRMs of PTB are not all equal and that the C-terminal RRM3-4 di-domain plays the critical role (Figure 7). This is reflected in the stronger RNA contacts (Figure 2), and by the highly detrimental effects of RNA binding (Figure 1) and di-domain packing mutations in RRMs 3–4 (Figure 6). The negligible effects of the same mutations upon affinity for *FAS* RNA (Table 1), suggest that the loss of function is mainly due to the disrupted architecture of PTB-RNA interactions. The lack of effect of the packing mutation upon PTB affinity for *FAS* RNA (Table 1), contrasts with more severe effects when the mutation was introduced into the RRM3-4 di-domain alone (13). Nevertheless, the packing mutation abolished all contacts of RRMs 3–4 of full length PTB with *FAS* RNA (Figure 3) and severely reduced splicing repressor activity (Figure 6). The apparent discrepancy between this result and the modest effects upon affinity for RNA might be related to the more stringent conditions of the probing experiment, which was carried out in nuclear extract where PTB contacts might be outcompeted by assembly of splicing complexes. PTB can repress splicing via interaction with U1 (45) or U2 snRNA (46). Repression of the *C-SRC* N1 exon involves interactions of the N-terminal RRMs 1 and 2 of PTB with stem-loop 4 of U1 snRNA bound at the 5′ splice site (45). If PTB acted in a similar fashion at *FAS* exon 6, we would have expected the RNA binding mutations of RRMs 1 and 2 to have led to a larger loss of function. These mutations were based upon RRM-CUCUCU interactions (11), and they disrupted RRM contacts with *FAS* RNA (Figure 3) as well as structured IRES RNA (18). It is possible that interactions with U1 snRNA are sufficiently distinct that they are not affected by the mutations used here. Nevertheless, taken at face value our results suggest that the mechanism of PTB action might differ between *FAS* and *C-SRC*.

Given the strength of the RRM3 contact at the URE6 silencer, one might expect that RNA binding mutations would be more deleterious in RRM3 than in any other RRM. However, RRM4 mutation had the most deleterious effect, similar to that of the packing mutant, even though it has the lowest intrinsic specificity of all four RRMs. This is consistent with an earlier report in which complete or partial deletion of RRM4 led to loss of repressor activity and contraction of the PTB footprint without loss of RNA binding (47). In stark contrast to the effect of mutations in RRM4, mutation of 5 of the 6 observed RNA contact sites of RRM4 (from YC to YU), reduced or abolished the RRM4-RNA contact, but had no effect upon splicing repressor activity (Figure 5). This suggests that RNA contact by RRM4 is important, but the precise site of contact is not. Furthermore, the lack of effect of RRM4 mutation upon contacts by RRM3 and other RRMs at URE6 demonstrates that these interactions are not sufficient for splicing repression. Thus, the architecture of the PTB *FAS* RNA interaction appears to be important for function, as originally suggested (11,13). Arguing against this, MS2 tethered PTB, but not PTB, can repress *FAS* exon 6 in which the URE6 has been replaced by MS2 sites (29). However, artificial recruitment systems have the potential to lead to the same regulatory outcome by different mechanisms.

One of the suggested functional consequences of the RRM3-4 di-domain packing is the creation of a high density of surface positive charge involving side-chains of amino acid residues in the linker and RRM4 (41). Consistent with this suggestion, mutation of linker residues K439 and K440, reduced contacts detected by cysteines in the linker and RRM4 and reduced the repressor activity of PTB (Figure 6). The functional effects were not as dramatic as the packing mutation, but other residues contributing to the positive patch could not be mutated either because they are involved directly in RRM4-RNA interactions (H457 and K485) or because they have additional roles in di-domain packing (11,42,43) (Supplementary Figure S3). Combined with the observation that contacts probed by N432 (CL) and E518 (C4) were always immediately adjacent (Figure 2), the results with the K439/440A mutant are consistent with the idea that RRM3-4 di-domain packing creates a more extended RNA binding surface for RRM4, leading to a more stable, albeit low-specificity, interaction. However, we cannot rule out the possibility that RRM3-4 di-domain packing has positive influences upon PTB function in addition to organization of the linker-RRM4 positive patch. Our results support the suggestion that the mode of RNA interaction by RRMs 3–4 might be critical for splicing repressor activity (11,13). Previous analyses of *FAS* exon 6 splicing indicated that PTB blocked a cross-exon interaction in which U1 snRNP at the 5′ splice site promoted U2AF65 binding at the upstream 3′ splice site (29). Our probing data suggest that a loop could form between the RRM3-URE6 contact and RRM4 contacts in either of the flanking introns, which might then obstruct the formation of the exon-definition complex.

There are interesting contrasts between the interaction of PTB RRMs 3–4 to *FAS* pre-mRNA and the viral IRESs. While RRM4 and linker contacts were all immediately adjacent in *FAS*, this was not the case in the IRESs (15–17). Possibly the highly structured nature of the IRES RNA means that the nucleotides immediately adjacent to the RRM contacts are not always available to contact the linker surface. Again this points to considerable flexibility in the way in which PTB can contact RNA. Another contrast is in the response to RRM mutations; poliovirus IRES was non-responsive to PTB if RRMs 1, 2 or 4 were mutated, but was insensitive to RRM3 mutation, while EMCV was sensitive to RRM 1 and 2 but not RRM 3 and 4 mutations. The RRM dependency therefore seems to vary between different substrates even when the same function is regulated. A final technical point of comparison is that our analyses with *FAS* pre-mRNA were carried out in nuclear extracts functional for splicing. In contrast, the IRESs could not be analyzed in reticulocyte extracts due to the high catalase activity, which consumes the H_2_O_2_ in the probing reactions.

We (D.C., C.W.J.S., and I.C. Eperon) previously attempted to model the interaction of PTB with individual substrate RNAs by searching for occurrences of linked motifs matching YCUN_1–6_CUN_3–8_YCU, which correspond to the contacts of RRMs 1–3, respectively (26). This approach was able to fit multiple PTB molecules onto the long pyrimidine tracts flanking *Tpm1* exon 3, and indicated that individual PTBs could bind in multiple alternative registers. However, a 5′-3′ linear order of RRM1-3 binding was assumed, which is not supported by the data presented here (Figure 2). Perhaps a more important caveat is that the RRM specificity was based on the observed interactions of PTB RRMs with a CUCUCU RNA ligand. Computational analysis of PTB CLIP targets revealed enrichment not only of expected pyrimidine triplets such as UCU, UUU, UUC, but also of triplets containing one or two Gs (9), suggesting that one or more of the PTB RRMs can recognise G-containing motifs (9). Analysis of the global sequence preferences of individual RRMs might be facilitated by our single cysteine PTBs. These could be modified with bifunctional reagents containing a sulfhydryl reactive group to link to the cysteine, coupled via a spacer arm to a photoreactive group. By incubating with complex pools of RNA, it might then be possible to carry out a variant CLIP experiment (48) to interrogate RNA contacts of individual RRMs within a multi-RRM protein, thereby revealing the inherent specificities of the individual RRMs.

## SUPPLEMENTARY DATA

Supplementary Data are available at NAR Online.

SUPPLEMENTARY DATA

## References

[1] Auweter S.D., Fasan R., Reymond L., Underwood J.G., Black D.L., Pitsch S., Allain F.H. (2006). Molecular basis of RNA recognition by the human alternative splicing factor Fox-1. EMBO J..

[2] Tuerk C., Gold L. (1990). Systematic evolution of ligands by exponential enrichment: RNA ligands to bacteriophage T4 DNA polymerase. Science.

[3] Ray D., Kazan H., Cook K.B., Weirauch M.T., Najafabadi H.S., Li X., Gueroussov S., Albu M., Zheng H., Yang A. (2013). A compendium of RNA-binding motifs for decoding gene regulation. Nature.

[4] Cereda M., Pozzoli U., Rot G., Juvan P., Schweitzer A., Clark T., Ule J. (2014). RNAmotifs: prediction of multivalent RNA motifs that control alternative splicing. Genome Biol..

[5] Zhang C., Lee K.Y., Swanson M.S., Darnell R.B. (2013). Prediction of clustered RNA-binding protein motif sites in the mammalian genome. Nucleic Acids Res..

[6] Keppetipola N., Sharma S., Li Q., Black D.L. (2012). Neuronal regulation of pre-mRNA splicing by polypyrimidine tract binding proteins, PTBP1 and PTBP2. Crit. Rev. Biochem. Mol. Biol..

[7] Sawicka K., Bushell M., Spriggs K.A., Willis A.E. (2008). Polypyrimidine-tract-binding protein: a multifunctional RNA-binding protein. Biochem. Soc. Trans..

[8] Perez I., Lin C.H., McAfee J.G., Patton J.G. (1997). Mutation of PTB binding sites causes misregulation of alternative 3′ splice site selection in vivo. RNA.

[9] Han A., Stoilov P., Linares A.J., Zhou Y., Fu X.D., Black D.L. (2014). De novo prediction of PTBP1 binding and splicing targets reveals unexpected features of its RNA recognition and function. PLoS Comput. Biol..

[10] Singh R., Valcarcel J., Green M.R. (1995). Distinct binding specificities and functions of higher eukaryotic polypyrimidine tract-binding proteins. Science.

[11] Oberstrass F.C., Auweter S.D., Erat M., Hargous Y., Henning A., Wenter P., Reymond L., Amir-Ahmady B., Pitsch S., Black D.L. (2005). Structure of PTB bound to RNA: specific binding and implications for splicing regulation. Science.

[12] Simpson P.J., Monie T.P., Szendroi A., Davydova N., Tyzack J.K., Conte M.R., Read C.M., Cary P.D., Svergun D.I., Konarev P.V. (2004). Structure and RNA interactions of the N-terminal RRM domains of PTB. Structure.

[13] Lamichhane R., Daubner G.M., Thomas-Crusells J., Auweter S.D., Manatschal C., Austin K.S., Valniuk O., Allain F.H., Rueda D. (2010). RNA looping by PTB: evidence using FRET and NMR spectroscopy for a role in splicing repression. Proc. Natl. Acad. Sci. U.S.A..

[14] Petoukhov M.V., Monie T.P., Allain F.H., Matthews S., Curry S., Svergun D.I. (2006). Conformation of polypyrimidine tract binding protein in solution. Structure.

[15] Kafasla P., Morgner N., Poyry T.A., Curry S., Robinson C.V., Jackson R.J. (2009). Polypyrimidine tract binding protein stabilizes the encephalomyocarditis virus IRES structure via binding multiple sites in a unique orientation. Mol. Cell.

[16] Kafasla P., Morgner N., Robinson C.V., Jackson R.J. (2010). Polypyrimidine tract-binding protein stimulates the poliovirus IRES by modulating eIF4G binding. EMBO J..

[17] Yu Y., Sweeney T.R., Kafasla P., Jackson R.J., Pestova T.V., Hellen C.U. (2011). The mechanism of translation initiation on Aichivirus RNA mediated by a novel type of picornavirus IRES. EMBO J..

[18] Kafasla P., Lin H., Curry S., Jackson R.J. (2011). Activation of picornaviral IRESs by PTB shows differential dependence on each PTB RNA-binding domain. RNA.

[19] Heilek G.M., Marusak R., Meares C.F., Noller H.F. (1995). Directed hydroxyl radical probing of 16S rRNA using Fe(II) tethered to ribosomal protein S4. Proc. Natl. Acad. Sci. U.S.A..

[20] Donmez G., Hartmuth K., Kastner B., Will C.L., Luhrmann R. (2007). The 5′ end of U2 snRNA is in close proximity to U1 and functional sites of the pre-mRNA in early spliceosomal complexes. Mol. Cell.

[21] Kent O.A., MacMillan A.M. (2002). Early organization of pre-mRNA during spliceosome assembly. Nat Struct Biol.

[22] Rhode B.M., Hartmuth K., Westhof E., Luhrmann R. (2006). Proximity of conserved U6 and U2 snRNA elements to the 5′ splice site region in activated spliceosomes. EMBO J..

[23] Gooding C., Roberts G.C., Smith C.W. (1998). Role of an inhibitory pyrimidine element and polypyrimidine tract binding protein in repression of a regulated alpha-tropomyosin exon. RNA.

[24] Wollerton M.C., Gooding C., Robinson F., Brown E.C., Jackson R.J., Smith C.W. (2001). Differential alternative splicing activity of isoforms of polypyrimidine tract binding protein (PTB). RNA.

[25] Gooding C., Edge C., Lorenz M., Coelho M.B., Winters M., Kaminski C.F., Cherny D., Eperon I.C., Smith C.W. (2013). MBNL1 and PTB cooperate to repress splicing of Tpm1 exon 3. Nucleic Acids Res..

[26] Cherny D., Gooding C., Eperon G.E., Coelho M.B., Bagshaw C.R., Smith C.W., Eperon I.C. (2010). Stoichiometry of a regulatory splicing complex revealed by single-molecule analyses. EMBO J..

[27] Cheng J., Zhou T., Liu C., Shapiro J.P., Brauer M.J., Kiefer M.C., Barr P.J., Mountz J.D. (1994). Protection from Fas-mediated apoptosis by a soluble form of the Fas molecule. Science.

[28] Cascino I., Fiucci G., Papoff G., Ruberti G. (1995). Three functional soluble forms of the human apoptosis-inducing Fas molecule are produced by alternative splicing. J. Immunol..

[29] Izquierdo J.M., Majos N., Bonnal S., Martinez C., Castelo R., Guigo R., Bilbao D., Valcarcel J. (2005). Regulation of Fas alternative splicing by antagonistic effects of TIA-1 and PTB on exon definition. Mol. Cell.

[30] Izquierdo J.M. (2012). Cell-specific regulation of Fas exon 6 splicing mediated by Hu antigen R. Biochem. Biophys. Res. Commun..

[31] Izquierdo J.M. (2010). Heterogeneous ribonucleoprotein C displays a repressor activity mediated by T-cell intracellular antigen-1-related/like protein to modulate Fas exon 6 splicing through a mechanism involving Hu antigen R. Nucleic Acids Res..

[32] Izquierdo J.M. (2008). Hu antigen R (HuR) functions as an alternative pre-mRNA splicing regulator of Fas apoptosis-promoting receptor on exon definition. J. Biol. Chem..

[33] Bonnal S., Martinez C., Forch P., Bachi A., Wilm M., Valcarcel J. (2008). RBM5/Luca-15/H37 regulates Fas alternative splice site pairing after exon definition. Mol. Cell.

[34] Amir-Ahmady B., Boutz P.L., Markovtsov V., Phillips M.L., Black D.L. (2005). Exon repression by polypyrimidine tract binding protein. RNA.

[35] Forch P., Puig O., Martinez C., Seraphin B., Valcarcel J. (2002). The splicing regulator TIA-1 interacts with U1-C to promote U1 snRNP recruitment to 5′ splice sites. EMBO J..

[36] Das R., Laederach A., Pearlman S.M., Herschlag D., Altman R.B. (2005). SAFA: semi-automated footprinting analysis software for high-throughput quantification of nucleic acid footprinting experiments. RNA.

[37] Laederach A., Das R., Vicens Q., Pearlman S.M., Brenowitz M., Herschlag D., Altman R.B. (2008). Semiautomated and rapid quantification of nucleic acid footprinting and structure mapping experiments. Nat. Protoc..

[38] Seliktar D. (2012). Designing cell-compatible hydrogels for biomedical applications. Science.

[39] Johnson N.L., Kemp A.W., Kotz S. (2005). Univariate Discrete Distributions.

[40] Vitali F., Henning A., Oberstrass F.C., Hargous Y., Auweter S.D., Erat M., Allain F.H. (2006). Structure of the two most C-terminal RNA recognition motifs of PTB using segmental isotope labeling. EMBO J..

[41] Maynard C.M., Hall K.B. (2010). Interactions between PTB RRMs induce slow motions and increase RNA binding affinity. J. Mol. Biol..

[42] Conte M.R., Grune T., Ghuman J., Kelly G., Ladas A., Matthews S., Curry S. (2000). Structure of tandem RNA recognition motifs from polypyrimidine tract binding protein reveals novel features of the RRM fold. EMBO J..

[43] Joshi A., Esteve V., Buckroyd A., Blatter M., Allain F.H., Curry S. (2014). Solution and crystal structures of a C-terminal fragment of the neuronal isoform of the polypyrimidine tract binding protein (nPTB). Peer J..

[44] Gooding C., Kemp P., Smith C.W. (2003). A novel polypyrimidine tract-binding protein paralog expressed in smooth muscle cells. J. Biol. Chem..

[45] Sharma S., Maris C., Allain F.H., Black D.L. (2011). U1 snRNA directly interacts with polypyrimidine tract-binding protein during splicing repression. Mol. Cell.

[46] Zheng X., Cho S., Moon H., Loh T.J., Oh H.K., Green M.R., Shen H. (2014). Polypyrimidine tract binding protein inhibits IgM pre-mRNA splicing by diverting U2 snRNA base-pairing away from the branch point. RNA.

[47] Liu H., Zhang W., Reed R.B., Liu W., Grabowski P.J. (2002). Mutations in RRM4 uncouple the splicing repression and RNA-binding activities of polypyrimidine tract binding protein. RNA.

[48] Witten J.T., Ule J. (2011). Understanding splicing regulation through RNA splicing maps. Trends Genet..

