# Supplementary material for: The organization of RNA contacts by PTB for regulation of FAS splicing
Source: Nucleic Acids Res. 2014 Jun 21;42(13):8605–20. doi: 10.1093/nar/gku519 (PMC4117754; doi:10.1093/nar/gku519)
Supplement: SUPPLEMENTARY DATA [file supp_42_13_8605__index.html]

The organization of RNA contacts by PTB for regulation of FAS splicing — SUPPLEMENTARY DATA 

# The organization of RNA contacts by PTB for regulation of *FAS* splicing

## SUPPLEMENTARY DATA

**Files in this Data Supplement:**

- SUPPLEMENTARY DATA
