# Supplementary material for: The organization of RNA contacts by PTB for regulation of FAS splicing
Source: Nucleic Acids Res. 2014 Jun 21;42(13):8605–20. doi: 10.1093/nar/gku519 (PMC4117754; doi:10.1093/nar/gku519)
Supplement: SUPPLEMENTARY DATA [file supp_gku519_nar-01000-r-2014-File009.pdf]

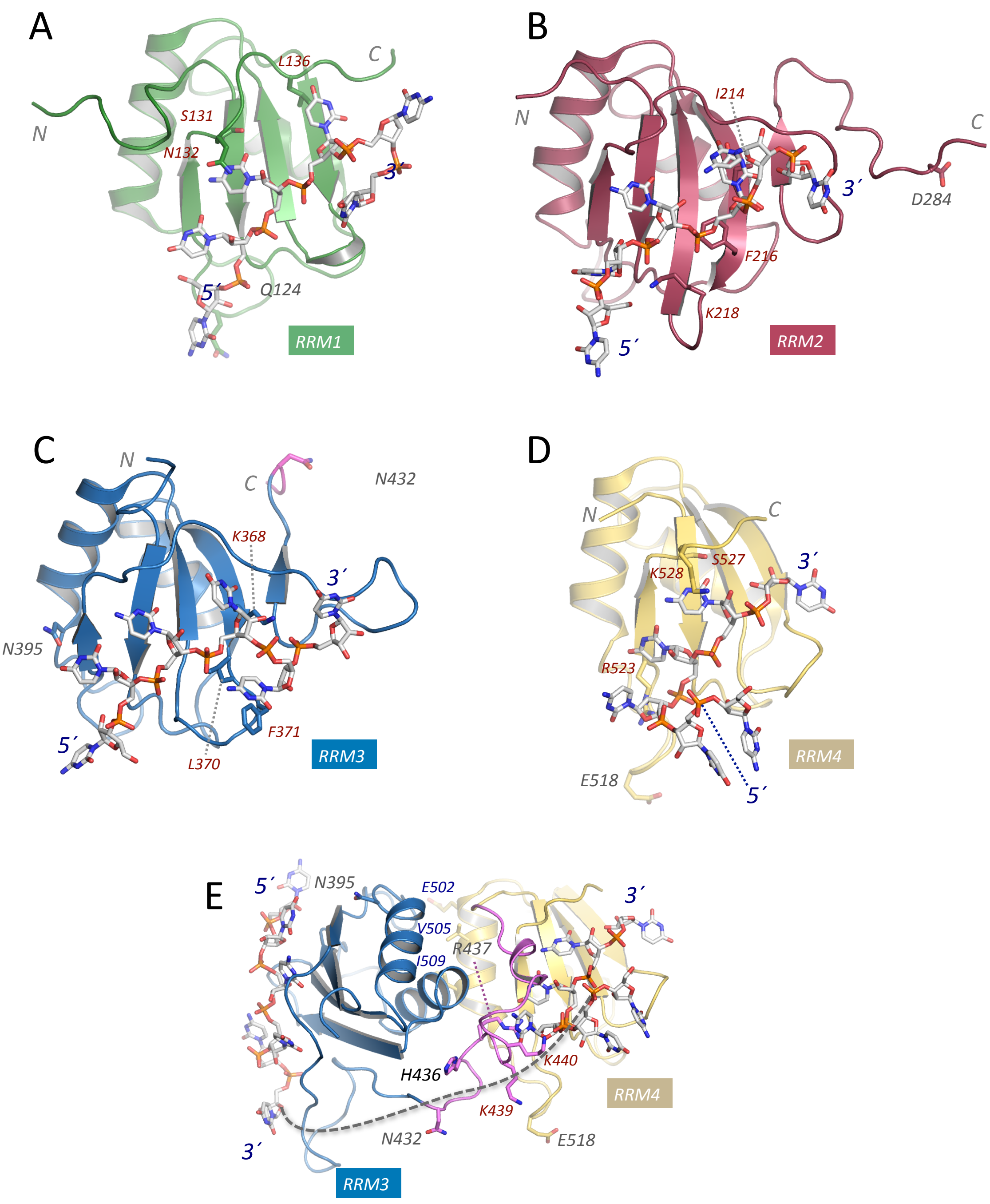

Supplementary Figure S1. RNA binding to the RRM domains of PTB and location of amino acids mutated to Cys for tethering of Fe(II)-BABE. Solution structures of (A) RRM1, (B) RRM2, (C) RRM3 and (D) RRM4 in complex with hexameric CUCUCU RNA oligomers (Oberstrass et al., 2005). The protein domains are shown schematically coloured using the same scheme as in Fig. 1. Bound RNA molecules are shown in a stick representation coloured by atom type (C – grey; O – red; N – blue, P – orange). Amino acid side chains mutated to Cys in each RRM (grey labels) or to abrogate RNA binding (dark red labels) or to disrupt RRM3-4 packing (dark blue labels) are also shown as sticks. (E) Solution structure of the PTB-34 di-domain showing the relative orientations of RRMs 3 and 4 and the bound CUCUCU oligomers (Oberstrass et al., 2005). The colour scheme is as in panels C and D. The grey dashed line indicated the possible path of a loop within a single stretch of RNA that is capable of binding simultaneously to RRMs 3 and 4. Key amino acid side chains discussed in the text are shown as sticks. These figures were prepared using PyMOL (The PyMOL Molecular Graphics System, Version 1.5.0.4 Schrödinger, LLC). Note, only a single conformation from the ensemble of structures deposited in the Protein Data Bank is shown (RRM1 – 2AD9; RRM2 – 2ADB; RRM3, RRM4 – 2ADC).

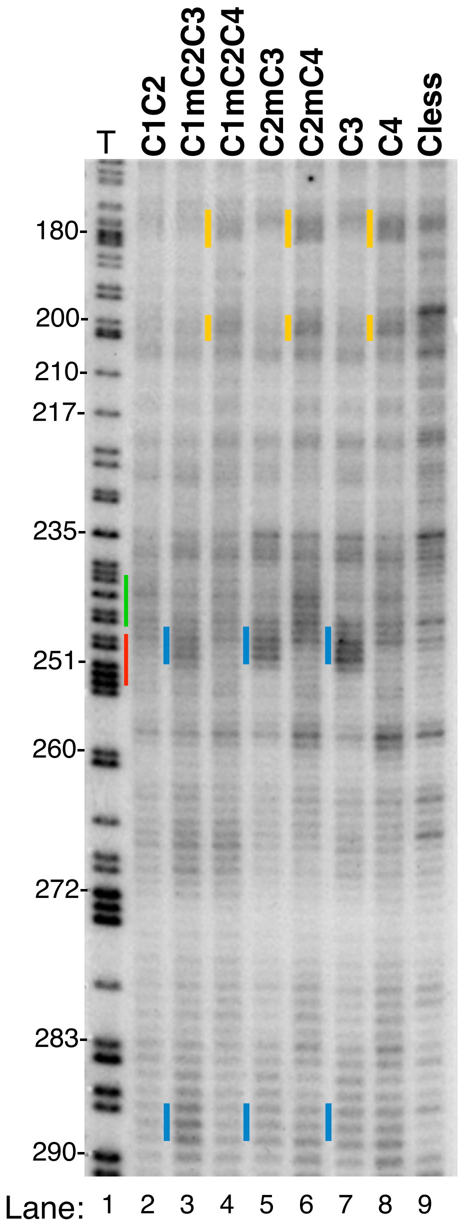

Supplementary Figure S2. Effects of the RNA-binding mutations in RRMs 1 and 2: Fe(II)-BABE Cys PTB1 mutants were used in directed hydroxyl radical probing assays. 0.1μM RNA were incubated with 0.9μM Fe(II)-BABE-PTB1 mutants. The RNA fragments produced after addition of H2O2 and ascorbic acid were analysed by primer extension. Cleavage sites are indicated by vertical lines on the left of the corresponding bands on the gel. The same colour-coding as in Figure 2 shows the different RRMs that produce the cleavages: green for RRM1, red for RRM2, blue for RRM3, and orange for RRM4. Lane 1 depicts a T sequencing ladder generated with the same primer.

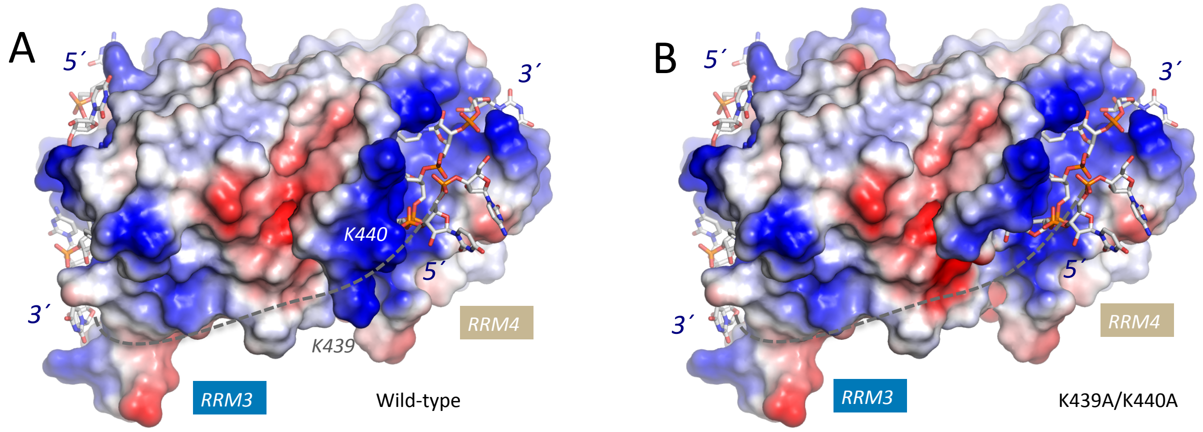

Supplementary Figure S3. Electrostatic surface of the PTB-34 didomain. The PTB-34 didomain is shown with a surface representation in the same orientation as in Supp. Fig. 1. The surface is coloured by the electrostatic potential (calculated using the vacuum electrostatics utility with PyMOL (Version 1.5.0.4 Schrödinger, LLC); positively and negatively charged surface features are coloured blue and red respectively. Model 8 from the ensemble of solution structures deposited in the Protein Data Bank (PDB ID 2ADC) was used for the calculation. The structures shown are (A) wild-type and (B) the K439/K440A double-mutant.
